# Pasireotide-Induced Shrinkage in GH and ACTH Secreting Pituitary Adenoma: A Systematic Review and Meta-Analysis

**DOI:** 10.3389/fendo.2022.935759

**Published:** 2022-07-01

**Authors:** Alessandro Mondin, Renzo Manara, Giacomo Voltan, Irene Tizianel, Luca Denaro, Marco Ferrari, Mattia Barbot, Carla Scaroni, Filippo Ceccato

**Affiliations:** ^1^ Department of Medicine (DIMED), University of Padova, Padova, Italy; ^2^ Endocrine Disease Unit, University-Hospital of Padova, Padova, Italy; ^3^ Department of Neuroscience (DNS), University of Padova, Padova, Italy; ^4^ Neuroradiology Unit, University-Hospital of Padova, Padova, Italy; ^5^ Neurosurgery Unit, University-Hospital of Padova, Padova, Italy; ^6^ Section of Otorhinolaryngology-Head and Neck Surgery, University-Hospital of Padova, Padova, Italy

**Keywords:** pasireotide, cushing, acromegaly, tumor volume, tumor size

## Abstract

**Introduction:**

Pasireotide (PAS) is a novel somatostatin receptor ligands (SRL), used in controlling hormonal hypersecretion in both acromegaly and Cushing’s Disease (CD). In previous studies and meta-analysis, first-generation SRLs were reported to be able to induce significant tumor shrinkage only in somatotroph adenomas. This systematic review and meta-analysis aim to summarize the effect of PAS on the shrinkage of the pituitary adenomas in patients with acromegaly or CD.

**Materials and methods:**

We searched the Medline database for original studies in patients with acromegaly or CD receiving PAS as monotherapy, that assessed the proportion of significant tumor shrinkage in their series. After data extraction and analysis, a random-effect model was used to estimate pooled effects. Quality assessment was performed with a modified Joanna Briggs’s Institute tool and the risk of publication bias was addressed through Egger’s regression and the three-parameter selection model.

**Results:**

The electronic search identified 179 and 122 articles respectively for acromegaly and CD. After study selection, six studies considering patients with acromegaly and three with CD fulfilled the eligibility criteria. Overall, 37.7% (95%CI: [18.7%; 61.5%]) of acromegalic patients and 41.2% (95%CI: [22.9%; 62.3%]) of CD patients achieved significant tumor shrinkage. We identified high heterogeneity, especially in acromegaly (I^2^ of 90% for acromegaly and 47% for CD), according to the low number of studies included.

**Discussion:**

PAS treatment is effective in reducing tumor size, especially in acromegalic patients. This result strengthens the role of PAS treatment in pituitary adenomas, particularly in those with an invasive behavior, with progressive growth and/or extrasellar extension, with a low likelihood of surgical gross-total removal, or with large postoperative residual tissue.

**Systematic Review Registration:**

https://www.crd.york.ac.uk/prospero/display_record.php?ID=CRD42022328152, identifier CRD42022328152

## Introduction

Pasireotide (PAS) is a novel somatostatin receptor ligand (SRL) with a high affinity for the somatostatin receptor (SSR) type 5 (1, 2). Somatotroph adenomas are usually responsive to first-generation SRLs (octreotide and lanreotide), as they are able to reduce growth hormone (GH) secretion through SSR type 2 (3). In the flow-chart of acromegaly treatment, PAS is suggested in case of resistance to first-generation SRLs, as SSR type 5 is also abundantly expressed in GH-secreting adenomas (3). A phase III study with PAS long-acting release (LAR) proved its efficacy in first-generation SRLs-resistant acromegalic patients after 6 months (4). In the extension study (Colao A et al.), 37% of patients achieved normalization of insulin-like growth factor 1 (IGF-1) and/or GH levels <1 µg/L, considering both those performing the extension treatment and those crossing over from the first-generation SRL-control group to the PAS LAR group. Nearly two-thirds of responses were achieved after at least 6 months of treatment. Up-titration of the dose from 40 to 60 mg monthly enriched the number of responders, suggesting that the PAS LAR effect may be both time- and dose-dependent (5). Concomitant improvement in signs and symptoms has also been confirmed in other series (6–9).

SSR type 5 is the predominant isoform in human corticotroph adenomas, since it is not down-regulated by high cortisol levels, as SSR type 2 does. Therefore, PAS is the only SRL available in patients with Cushing’s Disease (CD) (2). In a phase III study, subcutaneous (s.c.) PAS proved to be effective in normalizing urinary free cortisol (respectively in 13% and 25% of patients taking 600 µg or 900 µg bis-in-die for 12 months) (10), achieving significant clinical improvement (11). In the same clinical setting, PAS LAR showed similar efficacy and safety profiles (12). These benefits could be maintained for up to 5 years in an extension study (13, 14). In a recent meta-analysis, PAS treatment provided disease control in 44% of 522 patients with CD (15). Patients harbouring USP-8 mutations demonstrated an increased SSR type 5 expression in the corticotroph adenoma, increasing the likelihood of a positive response to PAS therapy (16). The safety profile of PAS is similar to that of first-generation SRLs, except for a significant worsening in glucose homeostasis (17).

Despite the normalization of hormonal excess, another goal of the medical treatment in GH-secreting pituitary adenomas is the reduction of the size of the adenoma (18). First-generation SRLs proved to be effective in achieving tumor shrinkage in acromegaly: Endocrine Society clinical practice guidelines suggested their role as primary therapy in poor surgical candidates and in those with extrasellar extension without chiasmal compression (18). Cozzi et al. reported in a large prospective cohort of acromegalic patients a significant Octreotide-induced tumor shrinkage in 82% of those receiving SRL as first-line treatment; most of them exhibited an early shrinkage with a progressive trend in reduction later on (19). A meta-analysis of 41 studies reported a significant tumor shrinkage in 50% of included patients (20). Data from the primary treatment with once-monthly lanreotide in surgical naïve patients demonstrated its efficacy in reducing tumor volume, achieving significant tumor shrinkage in 63% of them (21). Hypo-intensity on T2-weighted sequences at baseline magnetic resonance imaging (MRI) seems to predict tumor volume reduction during first-generation SRLs treatment (22). Regarding patients with CD, most patients presented a microadenoma, usually not aggressive or invasive: only in selected cases tumor shrinkage is an aim to achieve in patients with corticotropinoma.

As available data are scarce (or limited to selected studies), and the issue of pituitary adenoma shrinkage is of primary importance in the management of tumors that cannot be addressed through surgery, the aim of this systematic review and meta-analysis is to summarize available data regarding the effect of PAS on tumor size.

## Materials and Methods

**Figure 1:**
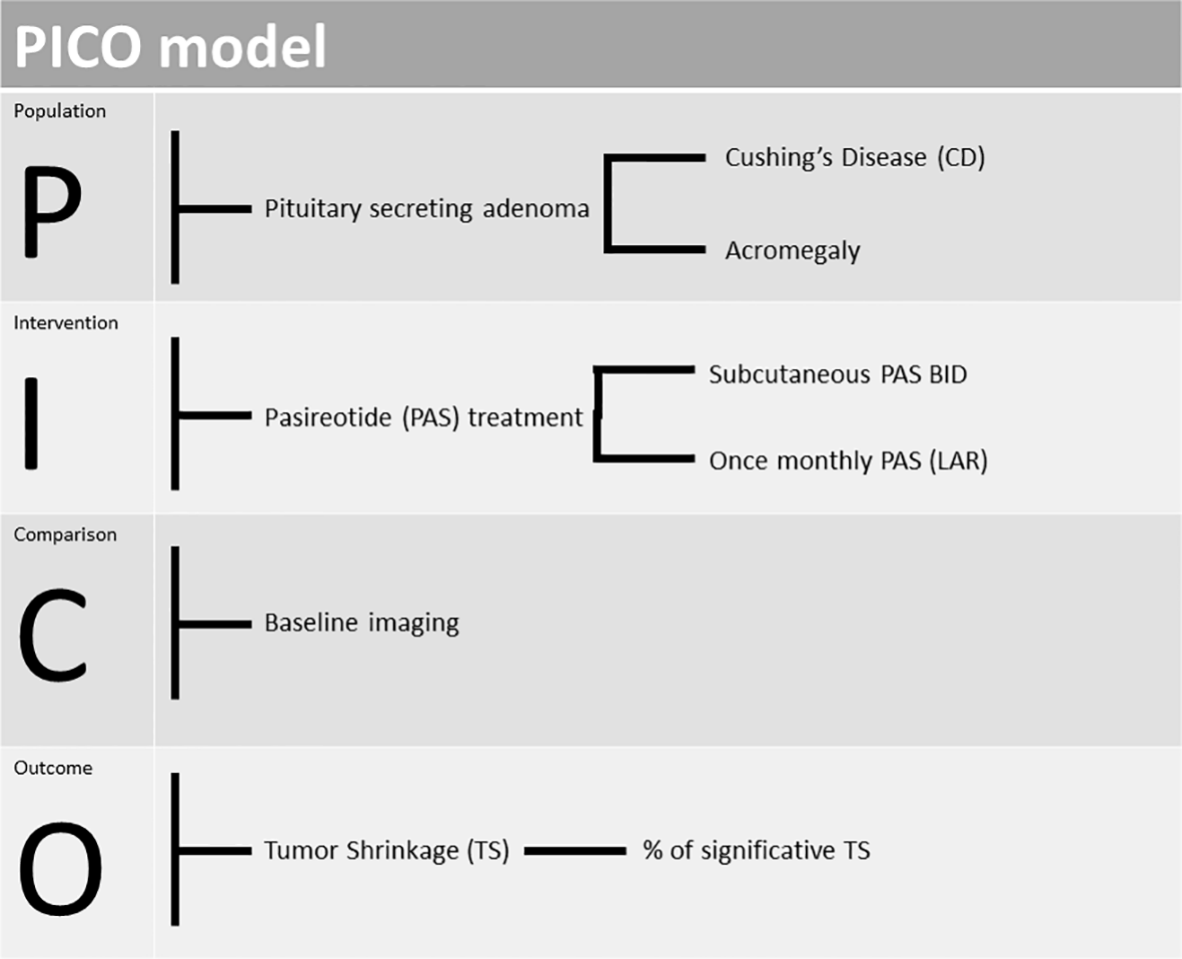
PICO (Population-Intervention-Comparison-Outcome) model design to our study.

We used the Population-Intervention-Comparison-Outcome (PICO) model to formulate the research questions for the systematic review (23), as summarised in **Figure 1**. The systematic review and meta-analysis were conducted and are reported according to the Preferred Reporting Items for Systematic Reviews and Meta-Analysis of Diagnostic Test Accuracy Studies (PRISMA-DTA) statement (24). We registered the protocol on the International Prospective Register of Systematic Reviews database (PROSPERO, https://www.crd.york.ac.uk/PROSPERO, number CRD42022328152).

### Search Strategy

An extensive Medline search was performed for the research question by two of the authors (F.C. and A.M.) independently, discrepancies were resolved by discussion. The literature search was performed up to January 2022, no language restriction was applied. Research included the following keywords: 1) (“acromegalies” [All Fields] OR “acromegaly”[MeSH Terms] OR “acromegaly”[All Fields]) AND (“pasireotide”[Supplementary Concept] OR “pasireotide”[All Fields]); 2) (“pituitary ACTH hypersecretion”[MeSH Terms] OR (“pituitary”[All Fields] AND “ACTH”[All Fields] AND “hypersecretion”[All Fields]) OR “pituitary ACTH hypersecretion”[All Fields]) AND (“pasireotide”[Supplementary Concept] OR “pasireotide”[All Fields]).

Inclusion and exclusion criteria were specified in advance and protocol-defined, in order to avoid methodological bias for *post-hoc* analysis. The searches were designed to select all types of studies (retrospective, observational, controlled, randomized, and non-randomized) conducted in patients with acromegaly or CD treated with PAS as monotherapy; the assessment of the proportion of significant tumor shrinkage was an inclusion criterion. Search terms were linked to Medical Subject Headings when possible. Keywords and free words were used simultaneously. Additional articles were identified with manual searches and included a thorough review of other meta-analyses, review articles, and relevant references. Consolidation of studies was performed with Mendeley Desktop 1.19.8.

### Study Selection

We included all original research studies conducted in adult patients that underwent PAS treatment used as monotherapy (s.c. bis-in-die and intramuscular once/monthly), that provided sufficient information about tumor size reduction during treatment. In case of overlapping cohorts of patients (as clinical trials with core and extension phases), we included only the extension study, in order to select those patients with measurable tumor shrinkage after long-term treatment. Local reports regarding patients involved in multicenter trials were excluded from the analysis, as they had been already considered in the larger series. Reviewers were not blinded to the authors or journals when screening articles.

### Data Extraction and Quality Assessment

Two authors (F.C. and A.M.) read the included papers and extracted independently relevant data, any disagreements were resolved by discussion. If data were not clear from the original manuscript, the authors of the primary study were contacted to clarify the doubts.

Contents of data extraction in the selected paper included: name of the first author, year of publication, setting (referral centre, academic hospital, mono- or multi-centric collection), type of treatment, its dose schedule and duration, pituitary imaging method during follow- up, the endpoint type regarding adenoma size analysis (i.e. primary vs exploratory). When data were reported for each patient or for subgroups, a global percentage of significant tumor shrinkage was calculated considering all subjects involved in the study.

To assess the risk of bias in the included studies, the critical appraisal tool from Joanna Briggs’s Institute (JBI) was adapted to evaluate those considered in our metanalysis (25). Among the items proposed, we selected the most appropriate to our setting: 1. Were the inclusion criteria clearly identified? 2. Were diagnostic criteria for acromegaly or CD well defined? 3. Were valid methods applied to evaluate tumor shrinkage? 4. Was the inclusion of participants consecutive and complete? 5. Was the reporting of baseline participants’ features (demographic and clinical) complete? 6. Was the report of the outcomes clear? 7. Was the report of demographics of the involved sites complete? 8. Was statistical analysis appropriate? For each aspect we assigned as possible choices of answer: yes, no or unclear.

### Data Synthesis and Analysis

A qualitative synthesis was performed summarizing the study design and population characteristics (age, male to female ratio, macro- to micro-adenoma ratio, prior treatments).

A random-effect model was used to estimate pooled effects. Forest plots for percentages of significant tumor size reduction were generated to visualize heterogeneity among the studies. In order to assess publication bias, despite the low number of articles considered, we performed funnel plot and asymmetry analysis adjusted for the low number of studies (an Egger’s regression test and a three-parameter selection model where two tailed p < 0.05 was considered statistically significant). The I^2^ test was conducted to analyze the heterogeneity between studies: an I^2^ >50% indicated a between-study heterogeneity.

Statistical analyses were performed with R: R-4.1.2 for Windows 10 (32/64 bit) released 2021-11-01 and R studio desktop RStudio Desktop 1.4.1717 for Windows 10 64 bit (R Foundation for Statistical Computing, Vienna, Austria, URL https://www.R-project.org/).

## Results

### Study Selection

**Figure 2:**
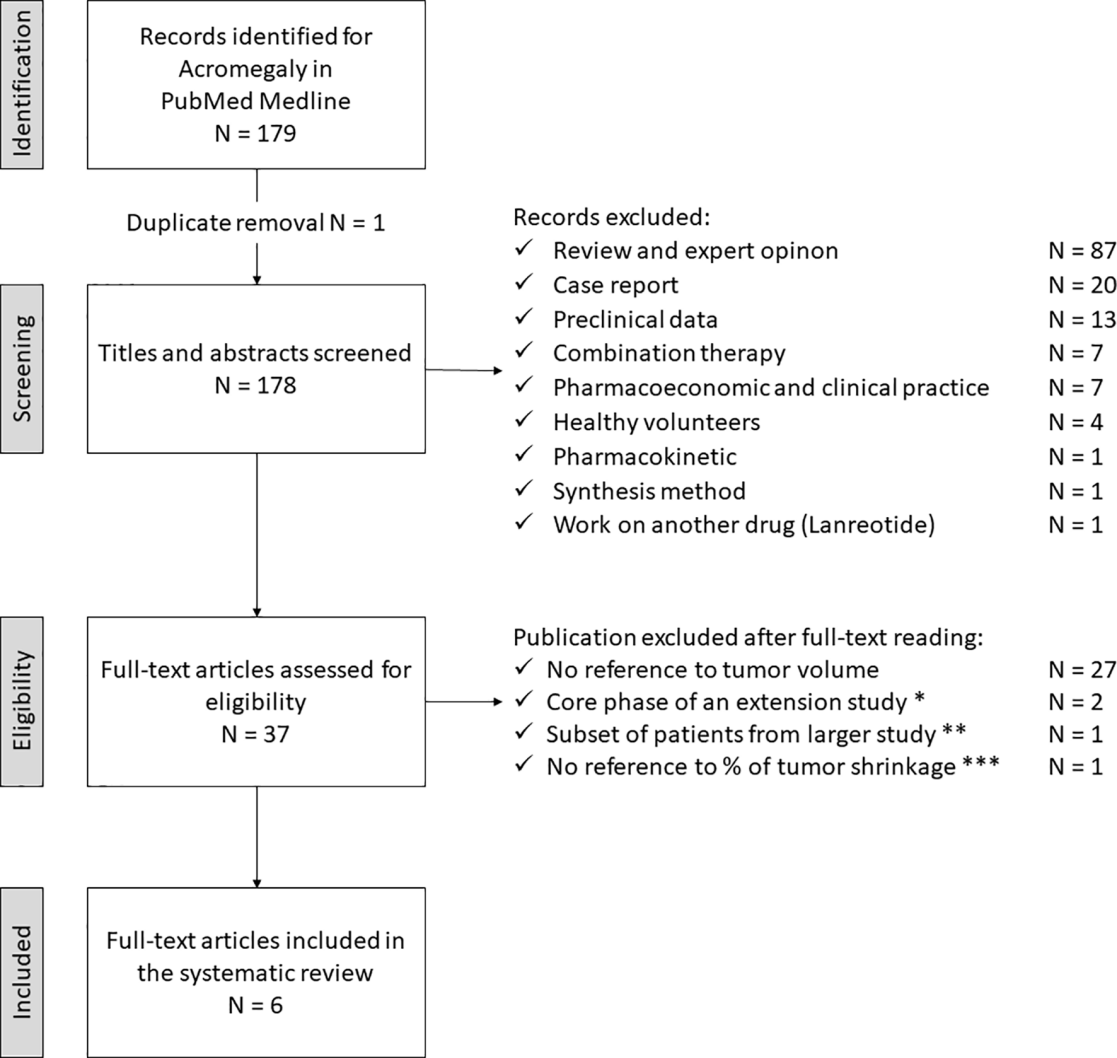
Search strategy for acromegaly. * Petersenn S, 2010 (PAS sc, phase II) and Colao A, 2014 (PAS LAR). ** Shimon I, 2015 (PAS LAR). *** Tahara S, 2019 (PAS LAR, phase II). PAS, pasireotide, sc, subcutaneous, LAR, long-acting release.

The study selection process for acromegaly is depicted in **Figure 2**. The electronic search revealed 179 articles, with one duplicate (N = 178). After the first screening, 141 articles did not meet the eligibility criteria and were discarded. The full-text examination of the remaining studies excluded additional 31 articles: 27 did not provide adequate data about tumor size, two represented the core phase of an extension study, another one referred to a subset of patients from a larger study, and the last one did not provide sufficient data about the percentage of tumor size reduction. Thus, six studies fulfilling eligibility criteria (reported in **Tables 1**, **2**), were selected for data extraction and analysis.

**Table 1:** Studies considered for the metanalysis in acromegaly.

Author, date	Design	Number		Prior treatments						Treatment	
		Number Recruited	Number for tumor size analysis	Naive	Surgery	RT	SRLs	CAB	PEG	Drug	Schedule
Petersenn S, 2013	Multicentric, prospective	30	29	13.3%	63.3%	20.0%	100.0%	na	na	PAS sc	200, 400 and 600 ug BID
Gadelha MR, 2014	Multicentric, prospective	130	81	0.0%	70.0%	3.9%	100.0%	32.3%	13.1%	PAS LAR	40 mg or 60 mg once monthly
Sheppard M, 2014	Multicentric, prospective	74	74	50.0%	50.0%	0.0%	0.0%	0.0%	0.0%	PAS LAR	40 mg once monthly
Bronstein MD, 2016	Multicentric, prospective	81	46	0.0%	43.0%	0.0%	100.0%	0.0%	0.0%	PAS LAR	40 mg once monthly
Lasolle H, 2019	Monocentric, prospective	15	9	0.0%	93.3%	26.7%	100.0%	26.6%	73.3%	PAS LAR	40 or 60 mg once monthly
Stelmachowska Banaś M, 2021	Monocentric, prospective	28	26	0.0%	96.0%	11.5%	100.0%	na	na	PAS LAR	40 mg once monthly

Dose titration was permitted in all studies. RT, radiotherapy, SRLs, somatostatin analogues, CAB, cabergoline, PEG, pegvisomant, na, not available, PAS, pasireotide, sc, subcutaneous, LAR, long active release, BID, bis-in-die.

**Table 2:** Studies considered for the metanalysis in acromegaly.

Author, date	Follow up		Tumor shrinkage as primaryendpoint	Criteria for tumor shrinkage	Macro:Micro-adenoma	M:F	Age	% of significant tumor shrinkage
	Mean duration	Exam						
Petersenn S, 2013	9 months (N = 9 up to 27 months)	MRI	No	Volume reduction >20%	na	14:16	45 (21 - 84)	58.60%
Gadelha MR, 2014	6 months	MRI	No	Volume reduction > 25%	na	57:73	45.5 (18 - 83)	24.05%
Sheppard M, 2015	25 months	MRI	no	Volume reduction >20%	na	36:38	46.5 (22 - 71)	74.70%
Bronstein MD, 2016	12 months	MRI	no	Volume reduction >20%	na	43:38	46 (25 - 86)	54.30%
Lasolle H, 2019	8 months (4 - 14)	MRI	no	Median height reduction	na	5:10	50 (27 - 67)	11.11%
Stelmachowska Banaś M, 2021	12 months	MRI	no	Volume reduction >20%	na	14:12	42.6 (23 - 67)	11.50%

M, male, F, female, %, percentage, N, number, MRI, magnetic resonance imaging, na, not available.

**Figure 3:**
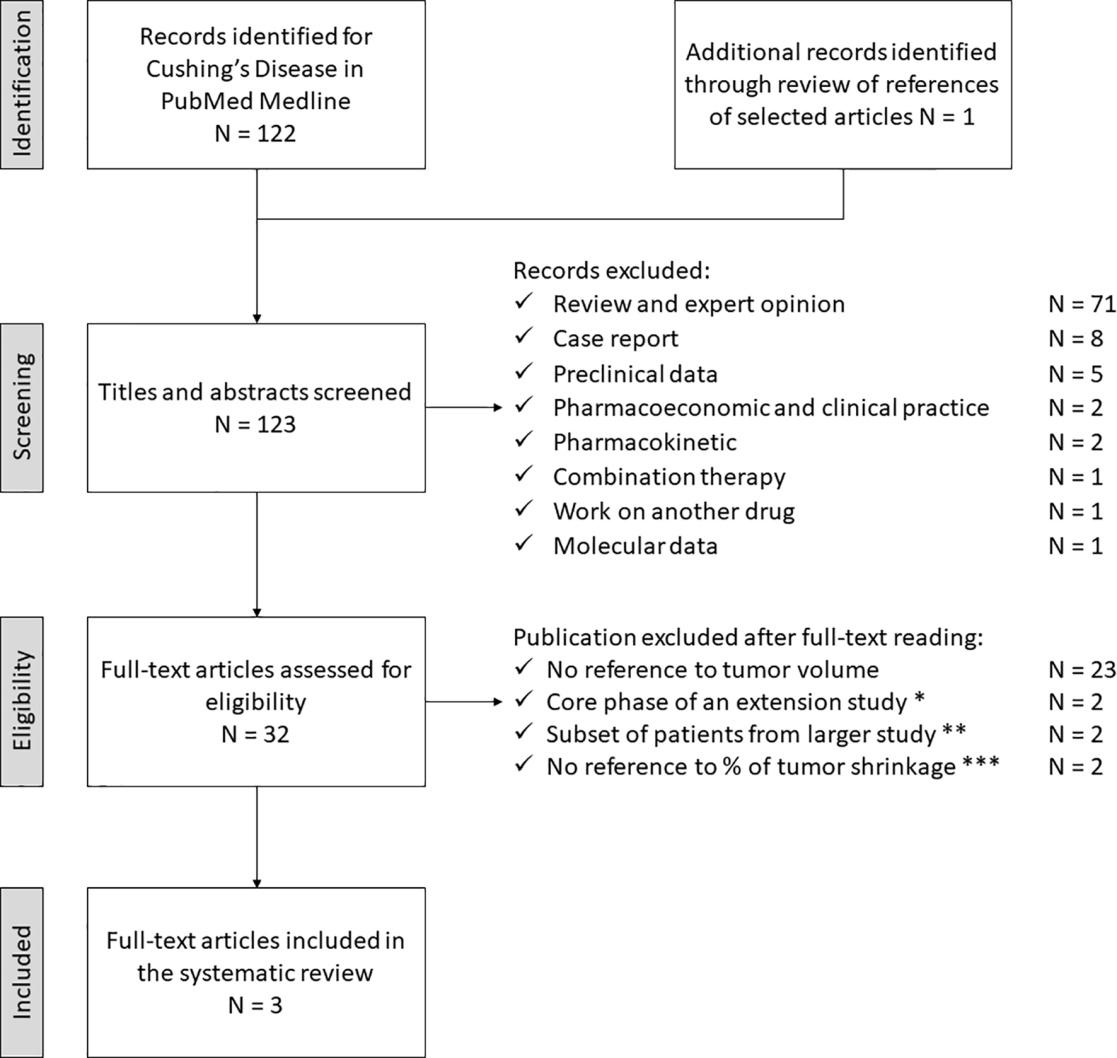
Search strategy for Cushing’s Disease. * Lacroix A, 2018 (PAS LAR, phase III) and Lacroix A, 2020 (PAS sc, phase III *post-hoc* analysis). ** Simeoli C, 2014 (PAS sc) and Colao A 2012 (PAS sc, phase III). *** Daniel E, 2018 (PAS sc and LAR) and Trementino L, 2016 (PAS sc). PAS, pasireotide, sc, subcutaneous, LAR, long acting release.

The study selection process for CD is depicted in **Figure 3**. The electronic search revealed 122 articles; an additional one had been included *post-hoc*, through reference analysis of selected articles (N = 123). After the first screening, 91 articles did not meet the eligibility criteria and were discarded. The full-text examination of the remaining studies excluded 29 more articles: 23 did not provide sufficient data on tumor shrinkage, two of them represented the core phase of extension studies, two referred to subsets of patients included in a larger study and two did not provide sufficient data regarding the percentage of tumor size reduction. Thus, three studies fulfilling eligibility criteria (reported in **Tables 3**, **4**) were selected for data extraction and analysis.

**Table 3:** Studies considered for the metanalysis in Cushing’s Disease.

Author, date	Design	Number		Prior treatments				Treatment	
		Number Recruited	Number for tumor size analysis	Naive	Surgery	RT	Other drugs	Drug	Schedule
Petersenn S, 2017	Multicentre, prospective	16	6	6.30%	87.50%	18.80%	56.30%	PAS sc	600/900 ug BID
Pivonello R, 2019	Multicentre, prospective	32	14	25%	78.12%	28.10%	62.50%	PAS sc	600 ug BID
Fleseriu M, 2019	Multicentre, prospective	81	14	na	82.70%	na	39.50%	PAS LAR	10/30 mg once monthly

Dose titration was permitted in all studies. RT, radiotherapy, na, not available, PAS, Pasireotide, sc, subcutaneous, LAR, Long active release, BID, bis-in-die.

**Table 4:** Studies considered for the metanalysis in Cushing’s Disease.

Author, date	Follow up		Tumor shrinkage as primary endpoint	Criteria for tumor shrinkage	Macro:Microadenoma	M:F	Age	% of significative tumor shrinkage
	Mean duration	Exam						
Petersenn S, 2017	60 months	MRI	No	Volume reduction > 25%	nd	2:14	44 (24 - 67)	50%
Pivonello R, 2019	6 months	MRI	No	Reduction in maximum diameter	6:8	7:25	47 (21 - 71)	21.40%
Fleseriu M, 2019	36 months	MRI	No	Volume reduction > 20%	4:10	20:61	39,7 ± 12,8	57.10%

M, male, F, female, %, percentage, MRI, magnetic resonance imaging. nd is na, not available.

### Study Characteristics

Four multi- and two mono-centric studies in patients with acromegaly were considered and analyzed, all presenting a prospective design. Tumor size analysis was not one of the primary endpoints in any of the considered studies; from an initial overall recruitment of 358 patients, only 265 were included for tumor size reduction analysis. Most patients had previously undergone different treatments (**Table 1**). All studies, except one, used PAS LAR, dose titration was allowed in all trials. Median follow-up ranged from 6 to 25 months; MRI was performed to evaluate tumor size reduction and the criteria for considering it significant was mainly based on tumor volume analysis, except for Lasolle H et al. which considered median height reduction (26). Data from the PAOLA study provided separate percentages of significant tumor shrinkage for PAS at 40 mg or 60 mg once monthly; considering that respectively 12 and 7 patients showed a reduction >25%, a significant shrinkage was reported in 19 out of 79 considered cases (24%) (4). Stelmachowska-Banás et al. described one patient with McCune-Albright’s syndrome presenting with pituitary hyperplasia, without a visible adenoma at MRI; as its pituitary volume decreased during treatment, the patient was included in the group with significant tumor shrinkage (27). No study provided information about macro- to micro-adenoma ratio. Data regarding age and male to female ratio are also reported in **Table 2**.

Three studies including patients with CD met the eligibility criteria (**Tables 3**, **4**); all of them presented a multicentre prospective design, recruiting 139 patients, most of them assuming PAS as a second-line treatment, after a surgical failure. For tumor shrinkage analysis, a subgroup of 34 patients was considered, taking s.c. PAS bis-in-die in two studies and PAS LAR in the third; in all cases titration was admitted. Tumor size analysis was a secondary endpoint in all three studies. Follow-up ranged from 6 to 60 months; tumor size assessment was performed with pituitary MRI. Only Pivonello et al. evaluated maximum diameter, instead of tumor volume changes (28). The population analyzed for tumor shrinkage mainly presented with a microadenoma. Data regarding age and gender are reported in **Table 4**. In the trial reported by Petersenn S et al., we arbitrarily fixed the criterion to define a significant tumor volume reduction (at least 25% of the baseline size of the pituitary adenoma), and the proportion of responders was calculated from the supplementary materials accordingly (3/6 = 50%) (13). Pivonello et al. separated patients exhibiting mild-moderate from those with severe hypercortisolism; we considered them together for tumor size analysis obtaining an overall proportion of significant size reduction of 21.4% (3 out of 14 subjects) (28).

### Risk of Bias

The evaluation of the risk of bias performed with the adapted JBI tool is reported in **Table 5**. All studies presented clear diagnostic and inclusion criteria, except that of Lasolle H et al. (26). Although all papers reported a valid tool for tumor shrinkage analysis (MRI), two of them did not analyse tumor volume and did not provide a clear definition of significant size reduction (26, 28). Regarding other items, the majority of the considered studies did not appear to present a clear source of bias.

**Table 5:** Evaluation of the risk of bias performed with the adapted Joanna Briggs’s Institute (JBI) tool.

Author, Year	1. Clear inclusion criteria?	2. Clear diagnostic criteria?	3. Valid method to evaluate tumor shrinkage?	4. Consecutive and complete inclusion of participants?	5. Complete reporting of baseline participants’ features?	6. Clear report of the outcomes?	7. Complete reporting of demographics of the involved sites?	8. Appropriate statistical analysis?
**Petersenn S, 2013**	yes	yes	yes	Yes	unclear	yes	yes	yes
**Sheppard M, 2015**	yes	yes	yes	Yes	yes	yes	yes	yes
**Bronstein MD, 2016**	yes	yes	yes	Yes	yes	yes	yes	yes
**Gadelha MR, 2014**	yes	yes	yes	Yes	yes	yes	yes	yes
**Lasolle H, 2019**	unclear	no	unclear	Yes	yes	yes	yes	yes
**Stelmachowska Banas M, 2021**	yes	yes	yes	unclear	unclear	yes	yes	yes
**Petersenn S, 2017**	yes	yes	yes	Yes	yes	yes	yes	yes
**Pivonello R, 2019**	yes	yes	unclear	unclear	yes	yes	unclear	yes
**Fleseriu M, 2019**	yes	yes	yes	Yes	unclear	yes	yes	yes

### Meta-Analysis

**Figure 4:**
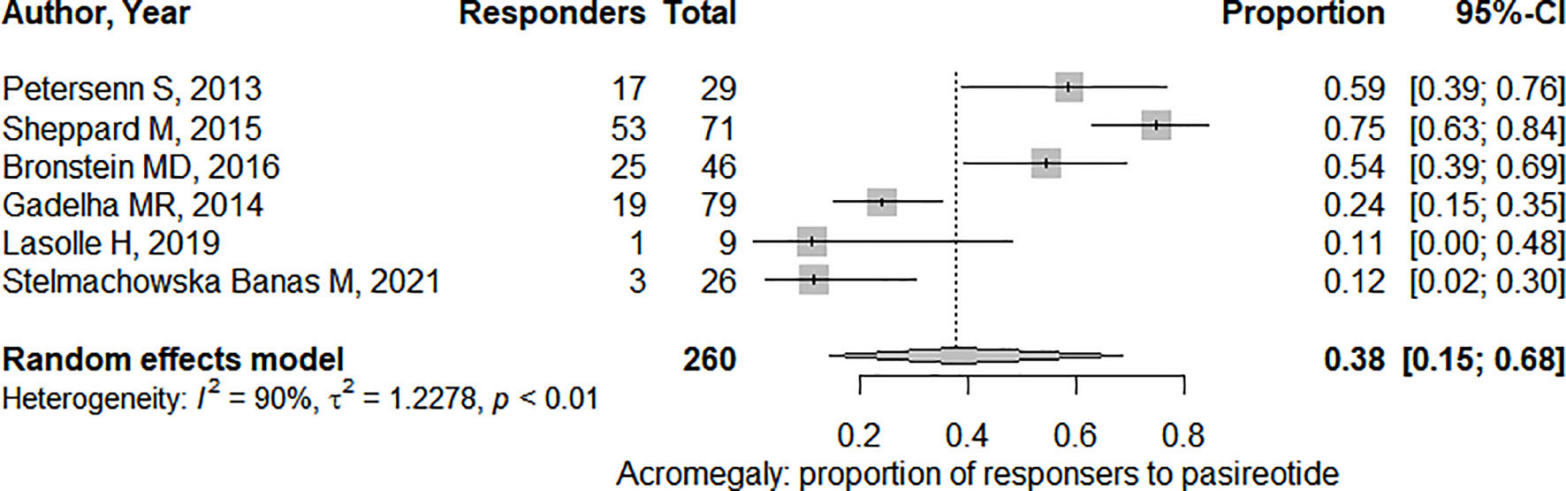
Pooled effect for the proportion of responders (i.e. presenting significant tumor shrinkage) in acromegaly. CI, confidence interval.

In the six studies considered for acromegaly, 37.7% (95%CI: [18.7%; 61.5%]) of patients demonstrated a significant tumor size reduction (**Figure 4**). As expected, heterogeneity in tumor reduction between studies was high (I^2^ = 90%). We attempted to address publication bias despite the low-number of studies (**Figure 6A**): Egger’s regression test did not indicate the presence of funnel plot asymmetry (intercept = -3.15 with 95%CI: [-10.17; 3.85], t = -0.883, p = 0.427) and the three-parameter selection model performed for p < 0.05 (and p < 0.1 as a sensitivity analysis) suggested absence of publication bias (28).

**Figure 5:**
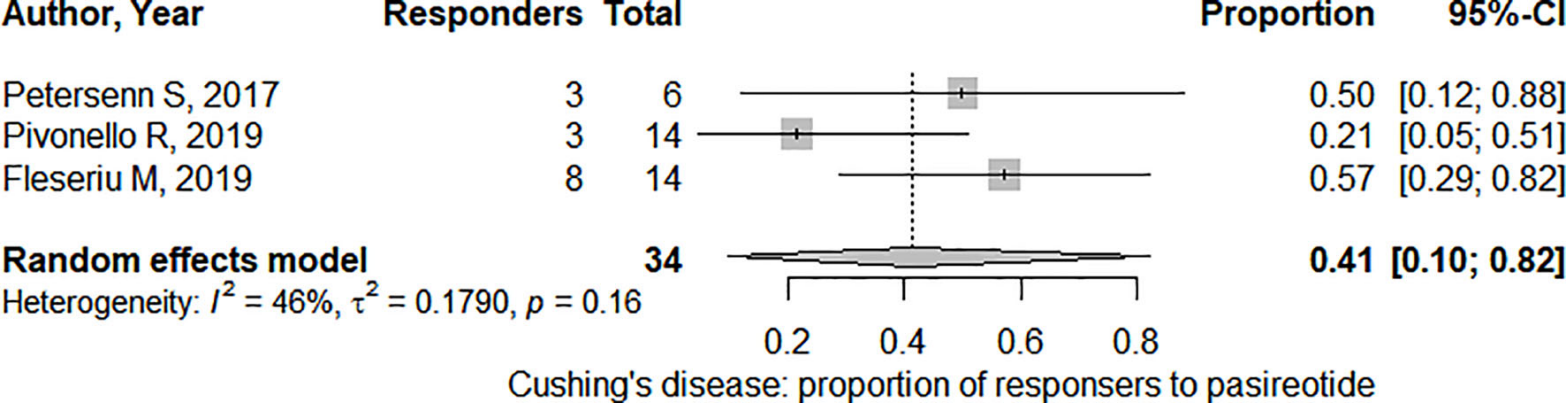
Pooled effect for the proportion of responders (i.e. presenting significant tumor shrinkage) in Cushing’s Disease. CI, confidence interval.

**Figure 6:**
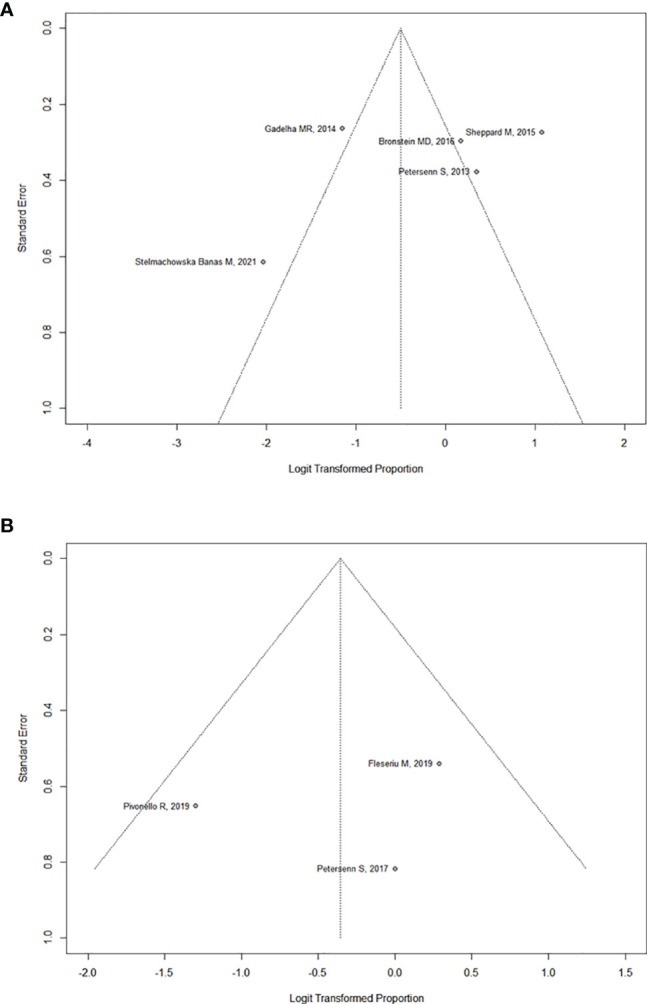
**(A)** Funnel plot assessing publication bias for Acromegaly. **(B)** Funnel plot assessing publication bias for Cushing’s Disease.

In the three studies considered for CD, 41,2% (95%CI: [22.9%; 62.3%]) of patients overall demonstrated a significant tumor size reduction (**Figure 5**). The heterogeneity in tumor reduction between the studies represented by I^2^ amounted to 47%. Publication bias analysis (**Figure 6B**) was performed using Egger’s regression test (intercept = -1.828 with 95%CI: [-14.53; 10.88], t = -0.282, p = 0.825) without evidence of asymmetry. The three-parameter selection model on the contrary could not be performed due to the small number of studies.

## Discussion

The biochemical efficacy of medical treatment with PAS in GH- or ACTH-secreting pituitary adenomas has been described in previous metanalyses for acromegaly (29, 30) and CD (15), the latter also exploring the clinical benefit. In addition to these reports, this meta-analysis shows that PAS treatment can induce an additional clinically significant tumor shrinkage in approximately 40% of patients.

### Acromegaly

Overall, PAS treatment provided tumor shrinkage in 37.7% of the considered patients. A previous metanalysis on octreotide in acromegaly provided a higher percentage of tumor size reduction (over 50%) (20). Nevertheless, since PAS treatment is usually considered as a second- or third-line treatment in the therapeutic flow-chart of acromegaly, the population recruited is mainly composed of patients with first-generation SRL-resistant somatotroph adenomas. This bias in recruited populations of acromegalic patients may explain this difference in the outcome. In a direct comparison, although PAS LAR seemed more effective in achieving biochemical control, both the SRLs, the first- and the second-generation types, achieved similar percentages of tumor shrinkage (6, 7). Moreover, in the crossover extension, the switch from octreotide to PAS was more effective than the reverse schedule, achieving a slightly higher percentage of further significant tumor shrinkage (8). Lasolle et al. reported that the expression of SSR type 5 and the granulation pattern are of limited value for the prediction of PAS responsiveness: 5 out of 9 somatotropinomas in their series were densely granulated (two did not respond to PAS), and the expression of SSR type 5 was modest in one controlled patient (26).

Other than SRLs, a further therapeutic option targeting the somatotroph adenoma is cabergoline, either as monotherapy in mild cases or as an add-on treatment for resistant adenomas (18). In a previous metanalysis, cabergoline in monotherapy resulted less effective than SRLs, achieving tumor shrinkage in about one third of the enrolled patients (31). It should also be mentioned that some studies reported an escape phenomenon from its treatment efficacy (32).

Data coming from the combination of PAS LAR and pegvisomant in acromegaly were not considered in our metanalysis, due to inclusion criteria and variable combination therapy of the two drugs (33). Since some cases of adenoma growth had been reported during pegvisomant use (34, 35), this combination therapy represents a rational approach, but tumor volume analysis is less reliable, given the purpose of our study. Despite concerns regarding tumor growth, pegvisomant effectiveness in acromegaly is well documented (18, 29), although the cost of this combination treatment can limit its applicability in real-life practice. Moreover, it is worth mentioning Coopmans and collaborators’ follow-up analysis, suggesting a PAS mediated anti-tumoral effect in acromegaly. During treatment, patients exhibited a significant increase in T2-weighted sequences signal at MRI; moreover, patients exhibiting this MRI characteristic in their adenomas showed a more evident decrease in IGF-1 levels, but not a similar pattern in reduction of pituitary adenoma size (36). This finding may be related to cell degeneration or tumor cell necrosis, without necessarily determining significant tumor size reduction. Further studies, probably with more data coming from histological reports, may be necessary to better understand these findings.

### Cushing’s Disease

Overall, PAS treatment provided significant tumor shrinkage in 41.2% of CD patients. Regarding pituitary-directed drugs, at this moment available for CD treatment, the efficacy of cabergoline has been proven *in vitro* studies, but its efficacy in clinical trials is still debated (15, 37). In a previous prospective study, cabergoline induced significant tumor shrinkage (defined as tumor volume reduction >20%) in 4 out of 20 (20%) of the patients recruited after 24 months (38). PAS is the only pituitary-directed treatment for this condition approved by Drug Agencies. Although few studies have been considered in this metanalysis, due to the strict inclusion criteria, PAS appears more effective in tumor size reduction versus cabergoline, resulting in a better choice in CD therapy when aiming to control the pituitary adenoma.

In contrast to acromegaly, the majority of CD patients present a microadenoma, suggesting that tumor size might be a less relevant issue during medical treatment, even if the “cure” of the disease may forecast the resolution of the adenoma. Besides, up to 30% of CD patients, depending also on MRI accuracy and neuro-radiologist’s expertise, may present with negative imaging that prevents any evaluation of tumor shrinkage (39). In spite of that, endocrinologists, not so infrequently, deal with aggressive corticotroph adenomas, characterized by invasive local growth and/or resistance to conventional therapies. This challenging entity often requires multidisciplinary expertise to suggest different approaches, including PAS treatment (40). It should be mentioned that some non-pituitary targeting drugs, as inhibitors of cortisol synthesis, have been associated with tumor growth, due to cortisol-ACTH negative feedback. In particular, during osilodrostat treatment in a phase III study, four recruited patients discontinued osilodrostat after a significant increase in tumor volume (two with micro- and two with macro-adenomas 41), and this growth had also been described during ketoconazole and mitotane treatments (42). Thus, it may be speculated that PAS could provide a rational approach as an combination treatment with steroidogenesis inhibitors. Moreover, after bilateral adrenalectomy, pituitary adenoma tumor size is of the utmost importance, as patients may be at risk of developing a progression of the adenoma, the so-called Nelson’s syndrome. In a prospective study from Daniel E et al., PAS proved to be also effective in this setting, reducing ACTH levels and stabilizing the residual tumor over a treatment period of 7 months (43). Further studies, with longer treatment observation, may reveal whether PAS may achieve significant tumor shrinkage in these patients, as suggested by previous case reports in literature (44, 45).

## Conclusion

The main limitation of our study resides in the scarce literature provided up to now (260 patients with acromegaly and 34 with CD), in the different therapy schedules and different criteria for tumor shrinkage in the selected study (largest tumor diameter vs a selected percentage of reduction). Moreover, in none of the study tumor reduction was one of the primary endpoints, and surgery was performed before PAS in most patients (78-88% of CD and 43-96% of acromegaly).

PAS is a novel compound, with a rising role in the treatment of secreting pituitary adenomas. Thus, this topic might be amplified with more data coming from further clinical studies, as real-life studies, possibly also addressing markers predictive of response to this treatment (e.g., expression of SSR type 2 and type 5 or somatic mutations in USP8 at tissue level of ACTH-secreting adenomas). Nevertheless, we can already state that PAS treatment is effective in reducing tumor size, especially in acromegaly. Our results strengthen the role of PAS treatment in somatotroph and corticotroph adenomas, especially when tumor volume is a relevant issue (i.e. tumor progression, extrasellar invasion) (18, 39), as a neoadjuvant treatment before surgery or as tailored treatment, alone or in combination, in persistent disease or when surgery is not feasible. Future research aiming to characterize markers predictive of response could help to identify optimal candidates for this treatment.

## Data Availability Statement

The original contributions presented in the study are included in the article. Further inquiries can be directed to the corresponding author.

## Ethics Statement

Informed consent was obtained from all subjects participating in the studies analyzed.

## Author Contributions

Authors involved contributed to research as reported: literature search (FC, AM), preparation of original draft (FC, AM, MB, LD), literature review (CS, FC, AM, MF), manuscript editing (CS, FC, AM, MB, LD, MF) and supervision (RM, CS). All authors approved the final version of the paper.

## Conflict of Interest

The authors declare that the research was conducted in the absence of any commercial or financial relationships that could be construed as a potential conflict of interest.

## Publisher’s Note

All claims expressed in this article are solely those of the authors and do not necessarily represent those of their affiliated organizations, or those of the publisher, the editors and the reviewers. Any product that may be evaluated in this article, or claim that may be made by its manufacturer, is not guaranteed or endorsed by the publisher.
